# Management of A Patient with Kommerrell's Aneurysm Causing Tracheal and Esophageal Compression

**Published:** 2009-06

**Authors:** B Ranjith Karthekeyan, Syama Sundar, Suresh Rao, Mahesh Vakamudi

**Affiliations:** 1 Associate Professor, Department of cardiothoracic anesthesiology, Sri Ramachandra Medical College and Research Institute, Porur, Chennai-400116; 2 Registrar, Department of cardiothoracic anesthesiology, Sri Ramachandra Medical College and Research Institute, Porur, Chennai-400116; 3 Professor, Department of cardiothoracic anesthesiology, Sri Ramachandra Medical College and Research Institute, Porur, Chennai-400116; 4 Professor & Head, Department of cardiothoracic anesthesiology, Sri Ramachandra Medical College and Research Institute, Porur, Chennai-400116

**Keywords:** Aneurysm, Trachea, Oesophagus, Tracheomalacia, Flexometallic tube

## Abstract

**Summary:**

Tracheal and esophageal compression is a well-recognized complication of aneurysms of the aortic arch. Most of the patients present with dysphagia and/or respiratory insufficiency. In the adult population a right-sided aortic arch is often asymptomatic unless aneurysmal disease develops. This usually occurs at the level of the take-off of an aberrant left subclavian artery and is known as a Kommerell's aneurysm. In spite of its rarity, this condition is clinically relevant because of the mortality associated with rupture, the morbidity caused by compression of mediastinal structures, and the complexity of surgery. In many cases, surgical resection of the aneurysm relieves the symptoms. We present a case in which tracheal compression and bilateral vocal cord palsy caused by an aneurysm arising from Kommerrell's diverticulum. The patient developed respiratory embrassement after extubation and was subsequently treated with continue positive airway pressure (CPAP) with a favorable result.

## Introduction

Tracheal and esophageal compression is a well-recognized complication of aneurysms of the aortic arch. Most of the patients present with dysphagia and/or respiratory insuffiency. In the adult population a right-sided aortic arch is often asymptomatic unless aneurysmal disease develops. This usually occurs at the level of the take-off of an aberrant left subclavian artery and is known as a Kommerell's aneurysm. In spite of its rarity, this condition is clinically relevant because of the mortality associated with rupture, the morbidity caused by compression of mediastinal structures, and the complexity of surgery^1^. In many cases, surgical resection of the aneurysm relieves the symptoms. We present a case in which tracheal compression and bilateral vocal cord palsy caused by an aneurysm arising from Kommerrell's diverticulum. The patient developed respiratory embrassement after extubation and was subsequently treated with continue positive airway pressure (CPAP) with a favourable result.

## Case report

A 30-year-old man was referred to our hospital with 10 day history of sudden onset of dysphonia and dysphagia. The patient had a road traffic accident 20 days before the development of symptoms. There was no history of stridor, wheezing, dyspnea, orthopnea or paroxysmal nocturnal dyspnea. Physical examination was normal except for the pulsatile mass at the right side of his neck. The blood pressure was 136/88 mm Hg and was equal in upper and lower limbs and heart rate was regular at 86 beats per minute.

**Fig 1:**
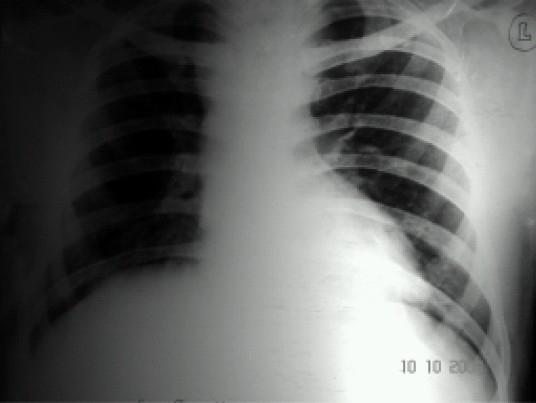
Chest x ray showing widened upper mediastinum

**Fig 2:**
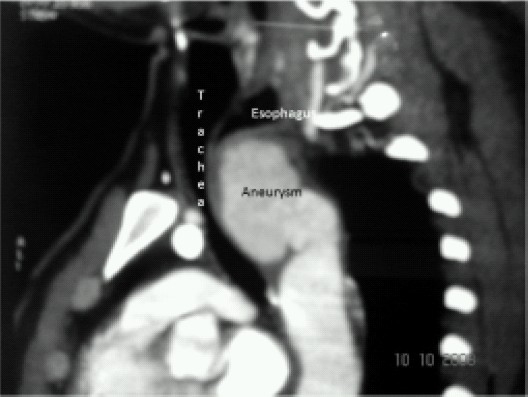
Computed tomographic scan showing compressed trachea and esophagus

Chest X-ray films showed widened upper mediastinum (Fig 1). Computed tomography demonstrated a saccular aneurysm (4.7cm × 6.8cm) arising from kommerrell's diverticulum of a right-sided aortic arch causing compression over the trachea and cervical esophagus (Fig 2). Angiography defined the ordering of the aortic arch branches as left common carotid, right common carotid, right subclavian and left subclavian arteries from proximal to distal. A pulmonary function test revealed the following values: forced vital capacity (FVC)of 2.73 litres (predicted63%), forced expiratory volume (FEV1)of 2.24 1itres and FEV_1_/FVC ratio of 100%. Indirect laryngoscopy revealed bilateral adductor palsy. Room air blood gas analyses were pH 7.47, PaO_2_ 84mmHg, and PaCO_2_ 32.2mmHg.

Preinduction monitoring included electrocardiogram, pulse oximetry, bispectral index and invasive blood pressure. The patient was induced with etomidate 20mg IV, midazolam2 mg IV, fentanyl 150 mcg IVand paralysed with rocuronium 60 mg IV after checking for ventilation. The maximum diameter of flexometallic endotracheal tube passed just beyond vocal cords was 6.5 mm. Inspite of tracheal compression beyond endotracheal tube intermittent positive pressure ventilation of the lung (IPPV) was instituted without difficulty. Left radial and right femoral artery was cannulated for monitoring the upper and lower limb pressures. Baseline values were heart rate of 90/mt, blood pressure of 130/90 mm of Hg and saturation of 100%. Right femoral vein was cannulated for central venous access. Post induction monitoring included urine output, temperature and central venous pressure. BIS was maintained around 40. Sevoflurane was used for maintenance of anesthesia. Fentanyl 50 mcgs, midazolam 1mg and vecuronium 1 mg was used whenever required.

**Fig 3:**
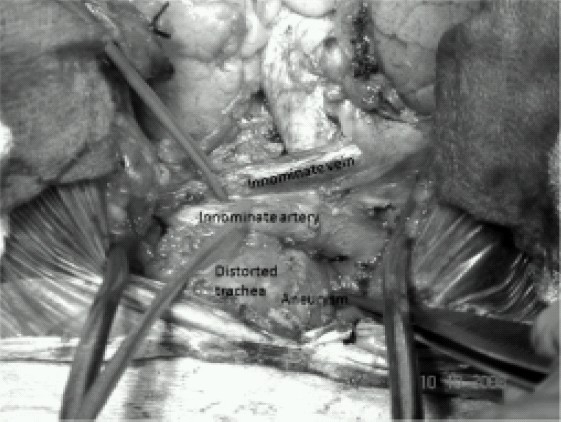
Intraoperative diagram showing innominate vein, innominate artery and aneurysm deviating the trachea

**Fig 4:**
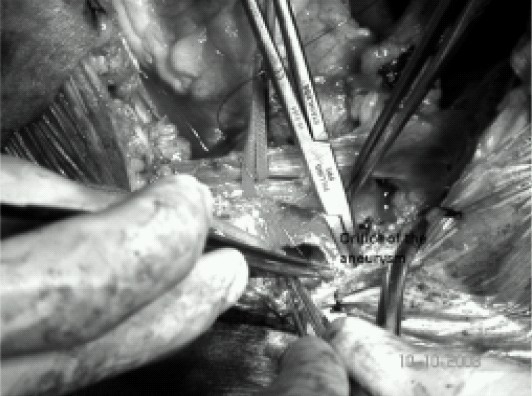
Intraoperative diagram showing the orifice of the sac being closed with Dacron patch.

The patient underwent exploration through a median sternotomy. After systemic heparinization, cardiopulmonary bypass (CPB) was established with cannulation to the left femoral artery and right atrium. Hypothermia was induced and circulation arrested at a nasopharyngeal temperature of 16°C for 16 minutes. At that time the head was packed in ice and surgical correction performed. The entire aneurysm was then excised relieving the posterior compression of the trachea and the esophagus (Fig 3). The orifice of aneurysm was located between the origin of right subclavian artery and left subclavian artery (Fig 4). Dacron patch was used to close the mouth of the aneurysm. Iatrogenic esophageal tear was repaired and feeding gastrostomy was done The systemic re-warming was completed and the patient was separated from cardiopulmonary bypass without any inotropic support. Total duration of cardiopulmonary bypass was 66 minutes. Just before weaning from cardiopulmonary bypass 6.5-mm flexometallic endotracheal tube was replaced with 7.5-mm flexometallic endotracheal tube. Protamine was administered and the sternotomy was closed after adequate hemostasis. The patient was shifted to intensive care unit with stable hemodynamics. The total blood loss was around 1 litre and urine output was adequate.

Patient was extubated the next day after ruling out tracheomalacia by leak test with deflating the tracheal cuff. After extubation, the patient developed respiratory distress for which he was kept on continuous positive airway pressure. Arterial blood gas showed a PaCO_2_ of 47 mm of Hg and PaO_2_ of 90 mmof Hg. The patient was unable to bring out the secretions due to bilateral adductor palsy. Vigorous physiotherapy helped to him to clear the secretions. He was also put on adrenaline, steroid and salbutamol nebulisation. The patient respiratory status improved and was discharged from the intensive care unit on the third postoperative day.

## Discussion

Right-sided aortic arch is a relatively rare congenital anomaly with an incidence of 0.1%^2^. The right-sided aortic arch results from persistence of the right fourth aortic arch and involution of the left. The right arch passes over the right main stem bronchus to the right of the trachea and esophagus^1^. Right-sided aortic arch may be asymptomatic. In infancy, symptoms are related to congenital heart anomalies or to compression of mediastinal structures such as the trachea or the esophagus. In adulthood, symptoms are more often the result of early atherosclerotic changes of the anomalous vessels, dissection, or aneurysmal dilatation with compression of surrounding structures causing dysphagia (dysphagia lusoria—“dysphagia by a trick of nature”), dyspnea, stridor, wheezing, cough, choking spells, recurrent pneumonia, obstructive emphysema, or chest pain^3^–^5^. Our patient had acute onset of dyspagia and dysphonia following road traffic accident.

The development of aneurysm usually occurs at the level of the take-off of an aberrant left subclavian artery and is known as a Kommerell's aneurysm. In spite of its rarity, this condition is clinically relevant because of the mortality associated with rupture, the morbidity caused by compression of mediastinal structures and difficulty in the airway management. The management of the aneurysm will depend on the anatomy, size and presence of a concomitant thoracic aortic aneurysm. Endoaneurysmorraphy is ideal for small Kommerell's aneurysms with a normal descending thoracic aorta, whereas an interposition graft is usually necessary for large Kommerell's aneurysms or Kommerell's aneurysms associated with an aneurysm of the descending thoracic aorta^1^. Our patient had a saccular aneurysm of size 4.7 × 6.8 cm and the orifice of the sac was closed with a dacron patch.

The anesthetic considerations in aortic arch aneurysm surgery are compression of the airway, massive blood loss, deep hypothermic circulatory arrest and tracheomalacia after extubation. Our patient in addition had bilateral adductor palsy. Tracheal intubation with tracheal deviation and compression is challenging. The distorted airway anatomy makes intubation difficult. Moreover, the induction of general anesthesia could be risky, because it may precipitate complete airway closure and make facemask ventilation and tracheal intubation impossible. So loss of control of the airway can occur at any stage of anaesthesia and consequences may be fatal. Its management poses special problems due to frequent involvement of the lower airways. Various measures described in the literature are tracheostomy, change of patient's position, splinting the trachea and main bronchus with armoured tube, endobronchial tube or double lumen tube, femoro-femoral cardiopulmonary bypass and helium-oxygen mixture. Another concern is tracheomalacia in these patients, which can complicate both intubation and extubation. Pressure on the trachea exerted by the neck mass could have caused necrosis to parts of the tracheal wall, which can lead to complete collapse of the airway with muscle relaxation^6^.

The problems faced by us was intubating with a smaller size endotracheal tube and respiratory distress after extubation. The first problem was managed by changing to a larger size when the patient was on cardiopulmonary bypass after the obstruction was relieved. The second problem was possibly due to adductor palsy with retained secretions, mucosal edema and mild respiratory obstruction although tracheomalacia was ruled out by deflating the tracheal cuff..It was managed by vigorous chest physiotherapy to bring the secretions out and with adrenaline, salbutamol and steroid nebulisation.

Careful preoperative evaluation of airway, being prepared to deal with an emergency of airway obstruction and massive blood loss, adequate analgesia and post operative management of tracheomalacia are essential to a successful outcome.
